# Advance care planning with people living with dementia: ethical considerations of physicians in the United States and the Netherlands

**DOI:** 10.1093/geronb/gbaf155

**Published:** 2025-08-21

**Authors:** Jingyuan Xu (须静媛), David R Mehr, Marieke Perry, K Taylor Bosworth, Kate McGough, Wilco P Achterberg, Hanneke Smaling, Jenny T van der Steen

**Affiliations:** Department of Public Health and Primary Care, Leiden University Medical Center, Leiden, The Netherlands; Department of Family and Community Medicine, University of Missouri, Columbia, Missouri, United States; Department of Geriatrics, Radboud University Medical Center, Nijmegen, The Netherlands; Department of Primary and Community Care, and Radboudumc Alzheimer Center, Radboud University Medical Center, Nijmegen, The Netherlands; Department of Family and Community Medicine, University of Missouri, Columbia, Missouri, United States; Department of Family and Community Medicine, University of Missouri, Columbia, Missouri, United States; Department of Public Health and Primary Care, Leiden University Medical Center, Leiden, The Netherlands; University Network for the Care Sector Zuid-Holland, Leiden University Medical Center, Leiden, The Netherlands; Department of Public Health and Primary Care, Leiden University Medical Center, Leiden, The Netherlands; University Network for the Care Sector Zuid-Holland, Leiden University Medical Center, Leiden, The Netherlands; Department of Public Health and Primary Care, Leiden University Medical Center, Leiden, The Netherlands; Department of Primary and Community Care, and Radboudumc Alzheimer Center, Radboud University Medical Center, Nijmegen, The Netherlands

**Keywords:** Ethics, Clinical decision-making, Palliative care

## Abstract

**Objectives:**

Advance care planning (ACP) is important but complex with people living with dementia. This study aims to explore ethical considerations of physicians around ACP for dementia in two high-income countries (the United States and the Netherlands).

**Methods:**

In this qualitative study, semistructured interviews were conducted with Dutch and American physicians from specialties that provide end-of-life care for people with living dementia. Their considerations regarding ACP for people living with dementia were solicited after short animation video vignettes on two approaches to ACP, one focused on concrete treatment orders, and the other on setting global care goals. Interview transcripts were analyzed using thematic analysis with elements of reflexive thematic analysis to identify ethical considerations.

**Results:**

Interviews with 50 Dutch physicians and 47 American physicians and 3 nurse practitioners generated three themes of ethical considerations: (1) Respecting the autonomy of the person with dementia, (2) Rationality as the basis for decisions and subsequent actions, and (3) Minimizing burden and suffering.

**Discussion:**

The complexity of ACP for people living with dementia is reflected in the challenges within each ethical consideration and the tensions between them, especially between autonomy and rationality. We recommend an approach to ACP that balances the ethical considerations, focusing on the values of the people living with dementia and allowing flexibility in future decision-making to take the current best interest of the person into account.

Advance care planning (ACP) is regarded as a crucial part of care for people living with dementia (Timmons et al., 2022), aimed at improving goal-concordant care and quality of life (Kelly et al., 2019). ACP is defined as “a process of communication about future care and treatment preferences, values and goals with the person with dementia, family, and the health care team, preferably with ongoing conversations and documentation” (van der Steen et al., 2024b). The process of ACP has evolved from focusing on documenting treatment orders, such as do-not-resuscitate orders, to including goals-of-care conversations (Im et al., 2024; van der Steen et al., 2024a). Physicians may take different approaches to ACP on this continuum, depending on personal preferences and the setting they work in (Flo et al., 2016; Smaling et al., 2023).

In both the United States and the Netherlands, ACP is recommended for people living with dementia (Fazio et al., 2018; van der Steen et al., 2023), and treatment refusals in advance directives are legally recognized (NIH National Institute on Aging, 2022; van der Steen et al., 2023). In the United States, ACP is covered by Medicare, which is a federal health insurance covering people with disabilities and those aged 65 years and above (the U.S. Centers for Medicare & Medicaid Services, n.d.). It often focuses on discussing and completing documents such as a living will, a health care power of attorney, and medical orders for life-sustaining treatment (NIH National Institute on Aging, 2022), facilitated by forms such as MOLST (Martin & McDonald, 2014) or dementia-specific directives (Gaster & Pope, 2024; Gaster et al., 2017). A variety of physician and advanced practice nursing specialties may initiate ACP discussions. In the Netherlands, ACP discussions can be initiated by the general practitioner or specialists in the hospital, but they are most often initiated and reviewed regularly by physicians specialized in caring for older adults (formal name of the specialty: “elderly care physician”) in nursing homes upon admission as they take over the (generalist) medical care from the GP (van der Steen et al., 2023). While physicians specialized in caring for older adults may also support GPs in consultations for older people in community-based practice, geriatricians’ practice is hospital-based. Common topics of ACP in the Netherlands include general care goals and concrete decisions such as hospital admission (Ter Brugge et al., 2022).

For people living with dementia, decreasing decisional capacity due to the illness and the importance of engaging family make ACP a complex and ethically challenging process for physicians (Keijzer-van Laarhoven et al., 2020). As physicians usually lead ACP conversations and provide treatment that may depend on decisions made during ACP, understanding their views about the process and how ACP should inform care can provide important insights on this challenging process. Previous studies mainly focused on more practical issues such as timing, resources, and challenges in assessing cognitive capacity (Perin et al., 2021; Tjia et al., 2023; Wendrich-van Dael et al., 2020) or ethical considerations that influence physicians’ willingness to engage in ACP (Keijzer-van Laarhoven et al., 2020). A broader examination of ethical considerations by physicians about the whole process of ACP, from initiation to activation of previously made decisions, will add a deeper understanding of ACP in this population.

Thus, this study aims to explore ethical considerations of physicians from two high-income countries with different practice patterns (the United States and the Netherlands) on ACP for people living with dementia. The results of the current study may guide future training and the development or refinement of healthcare policy.

## Method

### Study design

This qualitative study is part of the CONT-END (Attempts to CONTrol the END of life in people with dementia) project on end-of-life care for people living with dementia, which investigated the acceptability of four interventions for people living with dementia from the perspectives of people living with dementia, family caregivers, and physicians from six countries (Smaling et al., 2023). The four interventions investigated in the CONT-END project included two approaches of ACP, the use of monitoring technology at the end of life, and euthanasia. The current study only included the data on ACP from Dutch and American physicians and was guided by a reflexive approach to thematic analysis (Braun & Clarke, 2021). Data collection took place between July 2020 and December 2022. The study was registered at the Netherlands Trial Registration (NL7985) on August 31, 2019, and is currently available on the International Clinical Trial Registry Platform (NL-OMON26073). The study received ethics approval from the local Dutch medical ethical board (METC-LLD, NL72354.058.19, April 10, 2020) and the Institutional Review Board of the University of Missouri (2046522, December 3, 2021). All participants signed an informed consent form. Participants received gift cards for their participation (25 euros in the Netherlands, 100 dollars in the United States). The consolidated criteria for reporting qualitative research (COREQ) checklist was used to report the methods and results of this study (Tong et al., 2007).

### Participants

We aimed to include 50 physicians in each country through convenience sampling. Physicians practicing in a specialty that could include the provision of end-of-life care for people living with dementia were eligible, as were American nurse practitioners in these specialties if they assumed responsibility for medical treatment. Examples of specialties included family medicine, internal medicine, geriatric medicine, older adult care, palliative medicine, and neurology. In the United States, physicians and nurse practitioners were recruited, and informed of the study through the email lists of the American Geriatrics Society and the American College of Physicians—Missouri section, emails to selected practitioners at the University of Missouri School of Medicine, personal contacts of the authors, and snowballing. In the Netherlands, only physicians were approached via email through the networks of Leiden University Medical Center (LUMC), including the general practitioner network Extramural LUMC Academic Network (ELAN), the academic long-term care network University Network for the Care Sector South Holland (UNC-ZH), and the Specialist training program for care for older adults (SOOL). Potential participants were provided with additional information on request. After receiving signed informed consent, researchers scheduled an interview.

### Data collection

Participants were introduced to the subject of the interview by watching two short animated video vignettes on ACP, along with two other vignettes of the CONT-END project (Smaling et al., 2023). The vignettes on ACP described approaches focusing on either making concrete treatment decisions, such as resuscitation and hospital admission (Dutch: 3 min 21 s, English: 3 min 27 s), or getting to know the person and establishing important care goals such as maximizing comfort (Dutch: 2 min 52 s, English: 2 min 49 s). The ACP vignettes were played in a random order, one after the other (Smaling et al., 2023). After each video, participants answered questions on 1) the acceptability of the approach to ACP presented in the vignettes for people living with dementia, and 2) whether they were willing to use the approach upon request of the person with dementia or their family caregivers. After both ACP videos, participants expressed whether they preferred one of the approaches for their patients with dementia or for themselves if they developed dementia. They also explained their reasons and possible exceptions. The whole interview process was anticipated to take 50 min to complete. Answering questions about ACP took roughly half of this time. Interviewers included two psychologists experienced in qualitative research, a U.S. research assistant with a background in biology and psychology, and Dutch graduate students in medicine (two female, one male) and psychology (one female). The first author (X. J.) conducted interviews in both countries. Students were trained by the first author with extensive feedback. Most interviews were performed online via Zoom, except for two Dutch interviews, which took place via the telephone, and one face-to-face due to technical problems with Zoom or preference of the participants. Prior to the interview, participants also answered an online questionnaire about their demographics and work experience, which took around 10 min to complete.

### Data analyses

We used descriptive statistics, analyzed with SPSS 29 (IBM, 2024), to characterize the study sample and the acceptability of and preference for the presented ACP approaches in both countries. Interviews were transcribed verbatim without a member check (Morse, 2015). Thematic analysis was facilitated by Atlas.ti 23. The analysis resembles a codebook approach of thematic analysis, with elements of reflexive thematic analysis (Braun & Clarke, 2021). A codebook was developed to facilitate coding by multiple team members, which consisted of X. J., H. S., and graduate students in medicine (two female, two male) or psychology (one female). Most students who performed the interviews were also involved in coding. An initial codebook was developed after H. S. and X. J. independently coded three Dutch interviews. Each subsequent interview was coded by two researchers independently. Regular consensus meetings were held, and the codebook was fine-tuned and expanded throughout the process. Students without coding experience first coded a training set consisting of three interviews and were coached on the job by the first author. The coding of the American interviews started after all the Dutch interviews were coded. The coding was largely inductive, while the structure of the codebook was influenced by the interview questions, and some researchers kept the theoretical framework of acceptability by Sekhon et al. (2017) in mind while coding. After all coding was performed, the first author gathered similar codes into code groups and generated initial categories and themes together with M. P. and J. T. S. The themes were discussed and revised reiteratively with input from X. J., D. R. M., H. S., M. P., and J. T. S. They have all been involved in qualitative research and research in end-of-life with dementia. The authors represented the two countries and several relevant clinical fields, such as family medicine and palliative medicine.

### Positionality of authors

X. J. and H. S. are full-time researchers with degrees in psychology. D. R. M. is a family physician, geriatrician, and palliative care physician in the United States. M. P. is a general practitioner in the Netherlands. W. A. is a physician specialized in caring for older adults in the Netherlands. K. T. B. and K. M. are medical students in the United States. J. T. S. is a full-time researcher with a background in epidemiology. X. J., D. M., P.M., H. S, J. T. S., K. T. B., and W.A. have previous experience in qualitative research. The medical doctors in the team all have extensive experience conducting ACP with people living with dementia. The other authors have no direct experience in conducting ACP but have had regular conversations with people living with dementia through interviews about ACP in the context of research. Among the authors, X. J., D. R. M., and M. P. consider ACP focusing on important goals much more suitable for people living with dementia than ACP focusing on making concrete treatment orders. This may have influenced the way we interpreted the data.

## Results

Fifty Dutch physicians, 47 American physicians, and 3 American nurse practitioners participated in the study. The interviews lasted an average of 57 min (IQR 50–63). The sample characteristics are presented in Table 1. More than half of the participants were female. Most Dutch participants were physicians specialized in caring for older adults who mainly practiced at nursing homes or outpatient settings, although the specialties of the American participants were more diverse, with the great majority practicing at hospitals. Around half of the participants had training in palliative care after medical school. Most had cared for multiple people living with dementia at the end of life in the last year. The majority of the American participants practiced in the state of Missouri (*n *= 20, 40%) or New York (*n *= 10, 20%). All Dutch participants practiced in the province South-Holland. Forty-nine (98%) Dutch participants and 38 (76%) American participants were moderately or very comfortable talking about end-of-life with people living with dementia and their family caregivers. Forty-six (92%) Dutch participants and 49 (98%) American participants indicated that they would most strongly consider the wishes of the person with dementia, compared to wishes of the family or the physician, when considering future care goals for people living with dementia.

**Table 1.** Sample characteristics.

Characteristics	The Netherlands (*n *= 50)	United States (*n *= 50)
**Female, % (*N*)**	70 (35)	52 (26)
**Age in years, median (IQR)**	48 (34, 59)	33 (28, 45)
**Medical specialty,^a^ % (*N*)**		
** Family physician**	24 (12)	24 (12)
** Physician specialized in caring for older adults**	52 (26)	6 (3)
** Geriatrician**	2 (1)	2 (1)
** Palliative care physician**	0 (0)	10 (5)
** Nurse practitioners**	0 (0)	6 (3)
** Specialist in training**		
** Older adult care**	22 (11)	0 (0)
** Internal medicine**	0 (0)	14 (7)
** Other^b^**	2 (1)	18 (9)
** Other^c^**	0 (0)	8 (4)
**Place of practice,^d^ % (*N*)**		
** Home**	46 (23)	26 (13)
** Nursing home**	74 (37)	44 (22)
** Hospital**	8 (4)	82 (41)
** Hospice**	16 (8)	26 (13)
** Private**	0 (0)	18 (9)
**Received training in palliative care after basic medical training, % (*N*)**	42 (21)	54 (27)
**Years of experience caring for people with dementia, median (IQR)**	18 (4, 25)	4 (1, 11)
**Number of patients with dementia who died last year, % (*N*)**		
** 0**	8 (4)	18 (9)
** 1–4**	16 (8)	36 (18)
** 5–9**	36 (18)	16 (8)
** 10–19**	20 (10)	16 (8)
** 20 or more**	20 (10)	14 (7)
**Personal experience with family or friends having advanced dementia at the end of life, % (*N*)**	48 (24)	60 (30)

*Notes.*

a One physician in each country had more than one specialty.

b Examples of other specialties of physicians in training included for palliative care, neurology, and geriatrics.

c Other specialties included neurologist, internal medicine, emergency physician, and ophthalmologist.

d Physicians could practice in multiple locations.

Across both countries, there was strong agreement that both forms of ACP were acceptable for people living with dementia or upon request of the person with dementia or their family. Among the 50 Dutch physicians, 43–46 (86–92%) answered “yes” to these three questions for concrete treatment orders, and 48–49 (96–98%) found the important goals approach acceptable for people living with dementia or by request of the person with dementia or family. In the United States, the numbers were 43–49 (86–98%) and 48–50 (96–100%), respectively. In both countries, either approach was preferred by at least a one third of the participants, both for people living with dementia (concrete treatment orders: 17 (34%) in the Netherlands, 18 (36%) in the United States; important goals: 21 (42%) in the Netherlands, 23 (46%) in the United States) and for the physicians themselves if they had dementia (concrete treatment orders: 16 (32%) in the Netherlands, 20 (40%) in the United States; important goals: 26 (52%) in the Netherlands, 21 (42%) in the United States). The other physicians [8–12 (16–24%) in the Netherlands, 9 (18%) in the United States] stated that they did not have a preference.

### Ethical considerations

**Figure 1.**
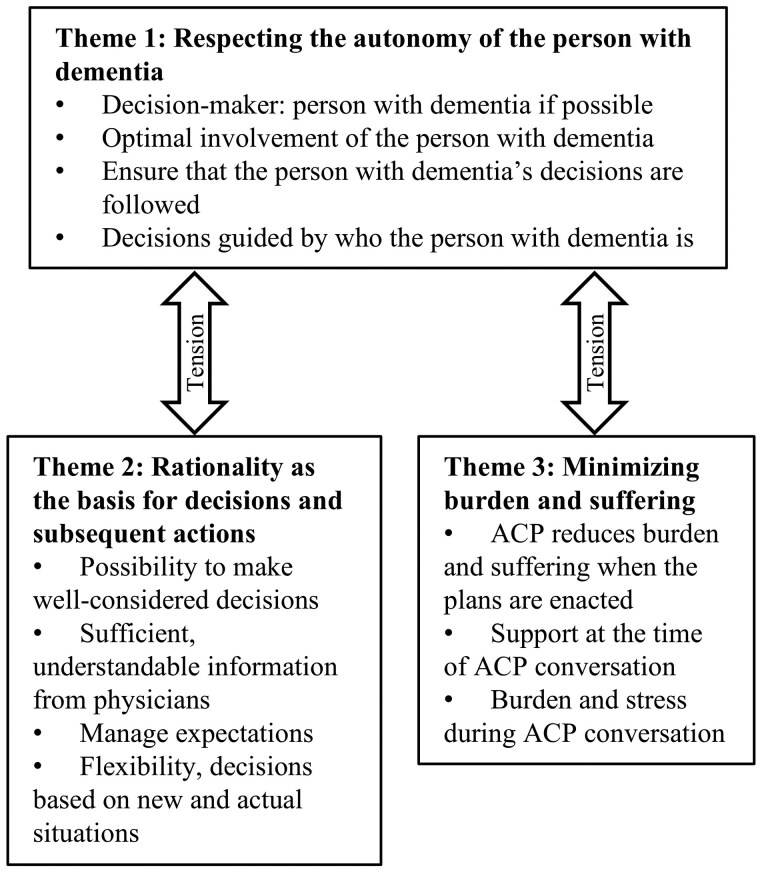
Ethical considerations of physicians in advance care planning for people with dementia. ACP = advance care planning.

Analysis generated three major themes representing the ethical considerations of professionals when conducting ACP with people living with dementia and when interpreting the decisions made during ACP. The themes and subthemes are presented in Figure 1.

#### Respecting the autonomy of the person with dementia

Many participants acknowledged that ACP is about the wishes of the person with dementia. Therefore, they considered supporting their autonomy to be one of the key considerations. Participants mentioned that whenever possible, it is essential to let people living with dementia be the decision-­makers. This means that their wishes should be heard and taken into account.

[I would’ve] preferred to hear from the patients themselves what they think about it [their medical decisions]. (Dutch physician in training to become a physician specialized in caring for older adults, aged 31 years)

This also included respecting the wishes of people living with dementia of not participating in an ACP conversation. When family caregivers were included in ACP conversations, some participants said to pay attention to whether they truly represent the interests of the person with dementia. A number of participants would only include families in ACP conversations in the presence of the person with dementia, or with their permission.

To maximize autonomy and decision-making for people living with dementia, participants suggested several ways to improve patient involvement. For example, physicians can initiate and encourage the conversation, make ACP easier by discussing important goals rather than concrete decisions, or let people living with dementia and their families prepare questions before the conversation.

It should be scheduled […] And so, they know they’re about what they’re going to be coming in to discuss when they come in. And that way, they can choose to bring whatever family member they want in with them. And they can think about their questions and read about it a little more before they come in. (the U.S. general practitioner, aged 30 years)

Some argued for a societal culture that favors ACP, which could be achieved by, for example, public education. A few participants acknowledged; however that ACP may not be acceptable for some people living with dementia from non-Western cultures.

If concrete decisions are made by the person with dementia during ACP, several participants mentioned that the wishes might not be honored by the family or physicians, but many participants found it important to ensure that these decisions are followed.

In one [of the approaches] you have a very concrete record of what they do and do not want. Then there is no room for discussion or interpretation afterwards. (Dutch physician in training to become a physician specialized in caring for older adults, aged 31 years)

A number of participants recognized that the ACP conversation itself helps to get all stakeholders on the same page and increases the chance of the decisions being followed.

So that the family knows how the patient feels. Otherwise, ehm, if the family doesn’t know, then the family might choose something different for the patient, even though the patient has things written down. (the U.S. geriatrician, aged 37 years)

Participants noted that the chance of the decisions of the person with dementia being followed would be enhanced by making the decisions specific, documenting them clearly, and communicating the decisions well with all professionals involved. Some American participants also emphasized the importance of legal aspects, such as assigning and involving a legal representative in the ACP and using a legal document.

Physicians also considered it important that medical decisions be guided by who the patient is. Some participants stated that knowing what is important for the patient was salient, and not to only focus on medical issues during ACP. Some suggested starting the ACP conversations with discussions about important goals, which will subsequently inform concrete decisions either during the ACP or in the future.

I think what it would give you is it gives you ehm, a better sense of ehm, what the patient’s values are so you can match future treatment decisions to those goals in, in a way that actually works better than just a, a checklist. (the U.S. geriatrician, aged 51 years)

#### Rationality as the basis for decisions and subsequent actions

Many participants emphasized that decisions and actions resulting from ACP need to be based on careful considerations and be clinically appropriate. Decisions were seen as well-considered if they are made beforehand, and by a person that has decisional capacity and not influenced by psychiatric conditions or strong emotions. Capacity of the person with dementia is a crucial requirement for many physicians to conduct ACP with them, especially when making concrete decisions, but also to establish important goals.

Ehm, no, I think if they’re not able to you know, repeat back or tell you those risks and those benefits [then ACP is not acceptable]. (the U.S. resident in cardiology, aged 27 years)

With dementia that is often still a bit of denial so to speak that you are actually that old and vulnerable. (Dutch general practitioner, aged 57 years)

Most of these participants did not mention the possible fluctuation of capacity. Many participants emphasized conducting ACP in an early stage, while others recognized the possibility of conducting the conversation as dementia progresses.

[…] some people just still understand a lot. Especially when the subject is the end of life. (Dutch general practitioner, aged 63 years)

To help people living with dementia make well-considered decisions, some participants stressed the importance of providing sufficient and understandable information during the ACP. They expressed the importance of giving clear explanations adapted to the capacity of the person with dementia.

I also think, very often it’s also very [good] to use examples, so it’s clearer to people oh this is what you mean. It’s the same with the resuscitation, where people often think, oh then you’re just going to press on my chest a few times and then I’ll go on with my life. Well of course it doesn’t work like that. So that’s how I explain, what resuscitation is and what it involves. (Dutch physician specialized in caring for older adults, aged 38 years)

Participants also suggested choosing an approach that is easier for the person with dementia. A few participants considered the less abstract discussion about concrete decisions to be easier. For many others, it was talking about important goals, which does not require an understanding of medical interventions and their consequences.

And when we have the patient or family explicitly saying yes, no to very particular medical interventions, ehm, it, it, it’s, it’s assuming that they know everything there is about those medical interventions which they don’t. They can’t. Ehm, I, I actually acknowledge even myself as a doctor, if I, if someone asked me if I wanted ECMO [extracorporeal membrane oxygenation], for instance, […] if I’m ever in a situation where I need that, I very much want someone who knows about ECMO to look at what matters to me in life and to say, is there a good chance that this treatment will allow these goals to be met. (the U.S. geriatrician, aged 33 years)

In addition to providing information, a number of participants argued that expectation management is part of ACP. This would prevent medically futile decisions based on unrealistic expectations. We observed cultural differences in perspectives on being directive. Dutch participants were more comfortable guiding the decisions. American participants were often more reserved in influencing ACP decisions.

For people with dementia who are already living in the nursing home, I usually say, well, I don’t think it’s helpful to resuscitate. And then I explain it, but then I actually more or less indicate that I have already made that decision. […] I don’t explain it as a choice. (Dutch physician specialized in caring for older adults, aged 37 years)

I would. At the end of the day, I would probably let the patient make that decision and treat them according to how they wish. (the U.S. family physician, aged 28 years)

Many participants stated that some flexibility is required to ensure rational and suitable decisions, as both a person’s condition and their preferences could change over time.

Yes, we did put cross there at the time [that the person still wanted to be taken to the hospital], but now they can’t even survive the ride to the hospital in an ambulance. (Dutch physician specialized in caring for older adults, aged 62 years)

Their needs and wishes may have changed completely. (Dutch physician specialized in caring for older adults, aged 62 years)

Some participants handled this problem of changing situations and wishes by making agreements less specific and more widely applicable, for example, by establishing goals rather than concrete treatment orders. Dutch participants, especially those with more experience, argued that physicians should be entitled to overrule decisions that no longer suit new situations. These experienced physicians also expressed more comfort in making decisions based on care goals, while many of their early-career colleagues expressed a wish to rely on clear decisions made in advance. American participants often mentioned that they would engage the ethics committee when previous decisions were absent or unsuitable, because, without specific direction, treatment could only be withheld in very rare cases.

In the U.S., you are presumed to want everything done until proven otherwise. […] I could tell you two examples in my career where the physicians decided, we are not going to offer CPR for this patient, even though we don’t have some decision maker telling us that. But that is here very rare. […] It’s the tradition, it’s the culture, it’s the medical legal system, it’s physicians being risk-averse. (the U.S. emergency physician and palliative care physician, aged 67 years)

#### Minimizing burden and suffering

According to many participants, one of the key reasons to conduct ACP is to minimize burden and suffering in the future.

In the U.S., we tend to do things to people at the end of life that are invasive, painful, and not helpful, and people need to impose restrictions to avoid unnecessary pain and suffering. (the U.S. emergency physician and palliative care physician, aged 67 years)

However, participants also expressed concerns over the possible burden and stress that the ACP conversation itself could induce. Therefore, they suggested several strategies to support the person with dementia and minimize the burden during ACP conversations, such as involving a supportive family caregiver.

Many participants were convinced that ACP will not only reduce future suffering and futile treatment for the person with dementia, but also reduce the stress and burden for decision-making of family caregivers and physicians.

It is very difficult for eh, a family member to say no to a treatment which is available, you know? […] this would be a time for the physician to gently remind them and say, you remember our conversation with Anna? What she said, she wanted comfort and if we’re going to do this, this, this, this could provide her pain or suffering. (the U.S. internist, aged 65 years)

They indicated that the decisions would be easier to make if the family caregivers and physicians felt the assurance that they were following the wishes of the person with dementia.

However, many participants recognized that people living with dementia and family caregivers may experience emotional distress during ACP conversations because of the sensitive topic of the end of life or because of conflicts and differences of opinion within the family. A few physicians said that they would support the people living with dementia and family as much as possible, by introducing the topic gently, including a supportive family caregiver, letting a physician who already has a trusting relationship with the person with dementia conduct the ACP session, and incorporating a calm and patient-­oriented attitude.

So I think, trying to approach ehm, death and morbidity with a calm and peaceful attitude sometimes helps patients realize that at some point, death is okay too. (the U.S. family physician, aged 29 years)

If the person with dementia was still likely to be stressed by the ACP conversation, some participants would choose to postpone it, only talk with the family, or forgo the conversation.

When people become very restless and tense when you talk about the end of life or about the future. Some people get very scared. […] So then, then I keep it very short. (Dutch physician specialized in caring for older adults, aged 37 years)

## Discussion

This study identified three key ethical considerations for Dutch and American physicians regarding ACP for people living with dementia: “respecting the autonomy of the person with dementia,” “rationality as the basis for decisions and subsequent actions,” and “minimizing burden and suffering.” Each of these considerations presents its own challenges, and they can conflict with each other. The main tension arises between autonomy and rationality. Prioritizing autonomy of the person with dementia requires strict adherence to all the concrete decisions made during ACP, which may conflict with the flexibility to make decisions based on the actual clinical situations that might arise. Strict observance of autonomy also potentially conflicts with the principle of minimizing suffering when prior decisions conflict with the current best interest. These tensions are also recognized in a review as a major barrier to physicians conducting ACP with people living with dementia (Keijzer-van Laarhoven et al., 2020).

The results suggest that ACP for people living with dementia is a complex quest for balance between conflicting ethical considerations. Autonomy, rationality, and minimizing suffering highly resemble the key principles of autonomy, beneficence, and nonmaleficence in clinical ethics (Varkey, 2021). The possible solution for the tension among them may also be comparable. According to Varkey (2021), the only ethically defensible option when autonomy conflicts with beneficence is to allow physicians to make medical decisions on behalf of a person when they lose capacity, taking into account their previously expressed values and current best interest. It is unjustifiable to focus solely on one of the principles, either by neglecting the patient’s wishes to prioritize beneficence or refraining from using the physician’s full knowledge to benefit the patient in order to maximize autonomy (Varkey, 2021). This balance between the ethical considerations therefore requires flexibility in carrying out advance decisions made during ACP. This flexibility needs to be recognized and discussed with people living with dementia and family caregivers to avoid false promises (Beck et al., 2017).

The necessity for flexibility requires a shift in the purpose of ACP from increasing goal-concordant care (Malhotra, 2023; Morrison et al., 2021; Sallnow et al., 2022) to enhancing overall well-being. Current evidence regarding whether ACP leads to goal-concordant care is inconsistent (Dixon et al., 2018; Kelly et al., 2019; Wendrich-van Dael et al., 2020), and we believe that this aim may not serve to meet the most important needs of people living with dementia. Research shows that only a small group of people with mild dementia expressed a need to make plans and take control of their lives (Hill et al., 2017). Instead, ACP aimed at goal-concordant care may primarily serve the needs of family caregivers who feel uncertain about making decisions for the person with dementia (Poole et al., 2018), and physicians who require documentation to mitigate legal risks associated with restricting or withdrawing life-sustaining treatments (Fleuren et al., 2020; Keijzer-van Laarhoven et al., 2020). The latter underscores the main difference observed in this study between the American and Dutch physicians in their willingness to make decisions based on the best interest of the person with dementia. This was also recognized in another interview study on decisions in people living with dementia and pneumonia, which found that physicians in the United States, unlike physicians in the Netherlands, were at times hesitant to decide in the patient’s best interest out of fear for disciplinary action that families could take against them (Helton et al., 2006). These needs may also explain some physicians’ preference for an ACP approach focused on making concrete treatment orders. For people living with dementia, the most pressing needs are support in coping with and accepting the disease, and respect (Miranda-Castillo et al., 2013; Shiells et al., 2020; van der Roest et al., 2007). These needs gradually shift to comfort and contact in later stages and the end of life (Fleuren et al., 2020; Shiells et al., 2020). New goals of ACP should therefore focus on facilitating well-being and adjustment for both people living with dementia (Dixon et al., 2018; Morrison et al., 2021), and their family caregivers (Malhotra et al., 2024). The aim of ACP should extend beyond improving care at the very end of life to the longer journey of “living with dying” (Abel et al., 2020). The first step in achieving this is to start ACP with discussions of current concerns and values of the person with dementia (van der Steen, 2024b).

Future research should investigate elements of ACP that help clarify values of the person with dementia and ways to document and communicate these values to best inform future care. Techniques to facilitate acceptance of future changes and unpredictability in the context of ACP warrant exploration. Since medical personnel have reported challenges in providing sufficient psychological support for people living with dementia (Tetrault et al., 2023), training could be developed or part of ACP could be delegated to coworkers, such as social workers, chaplains, and psychologists, who have more expertise in discussing values and facilitating adjustment (Hickman et al., 2023). Policy can be modified to protect physicians from legal risks when making in-the-moment decisions, especially when concluding a treatment is futile for the patient. Furthermore, studies should explore the needs of physicians who prefer making concrete treatment orders in ACP, and how these needs can be addressed within an ACP approach focusing on the discussion of values. Finally, the perspectives of other stakeholders in ACP, such as people living with dementia, family, and other healthcare professionals, should also be investigated and compared to the findings of the current study.

A strength of this study was the inclusion of physicians from diverse specialties across two countries, who shared their rich experiences and various perspectives. Soliciting their opinions with two ACP vignettes facilitated the expression of detailed personal experience and reflections on different approaches to ACP with people living with dementia. The rigor of the study was further enhanced by the reflexivity and triangulation of researchers with extensive clinical and research experiences in both countries. A limitation was that physicians with an interest in palliative care may be more likely to participate. This might explain the high acceptability of ACP among the participants. The difference in work experience and work setting between participants from the two countries may partly explain the more directive role that Dutch physicians take in ACP. Furthermore, the participants from the United States practiced in a few states. Generalization of their views to physicians working in other states should be done with caution. Lastly, due to their professional training and experience, participants in this study may have primarily considered medical care and the physical domain of well-being. However, the end-of-life care should be multidisciplinary, and attention should also be paid to psychological, social, and spiritual well-being in ACP (Fetherston et al., 2018).

In conclusion, Dutch and American physicians illustrated three themes representing ethical considerations that play significant roles in ACP for people living with dementia. ACP remains a complex process that requires weighing and balancing of ethical principles. We advocate for an ACP approach centered on the discussion of values of the person with dementia while allowing for a certain degree of flexibility in future decision-making.

## Data Availability

The data of this study are not publicly available due to privacy concerns. The study was preregistered at the Netherlands Trial Registration (NL7985) on August 31, 2019, and is currently available on the International Clinical Trial Registry Platform.
